# Towards a name change of schizophrenia: Positive and Negative Symptoms Disorder (PND)

**DOI:** 10.1007/s00406-025-02018-8

**Published:** 2025-05-19

**Authors:** Stefan Leucht, Deniz S. Yurtseven, Josef Priller, John M. Davis, Jim van Os, Iris E. C. Sommer

**Affiliations:** 1 Department of Psychiatry and Psychotherapy, School of Medicine and Health, Technical University of Munich, TUM University Hospital Rechts der Isar, Ismaningerstrasse 22, 81675 Munich, Germany; 2 German Center for Mental Health (DZPG), Partner Site Munich/Augsburg, Germany; 3 Department of Psychiatry, UMC Utrecht Brain Center, University Medical Center Utrecht, Utrecht University, Utrecht, the Netherlands; 4 Department of Psychosis Studies, Institute of Psychiatry, Psychology & Neuroscience, King’s College London, London, UK; 5 Neuropsychiatry and Laboratory of Molecular Psychiatry, Charité – Universitätsmedizin Berlin and DZNE, Berlin, Germany; 6 Centre for Clinical Brain Sciences, UK Dementia Research Institute at the University of Edinburgh, Edinburgh, UK; 7 Psychiatric Institute, University of Illinois at Chicago (Mc 912), 1601 W. Taylor St., Chicago, IL 60612 USA; 8 Maryland Psychiatric Research Center, Baltimore, MD USA; 9 Center for Clinical Neuroscience and Cognition, University Medical Center Groningen, Groningen, Netherlands

## Abstract

We propose renaming schizophrenia to Positive and Negative Symptoms Disorder (PND) to reduce stigma, enhance clarity, and align with current diagnostic criteria. The term reflects core symptom domains, is translatable, ICD compatible, and avoids misleading associations. If cognitive deficits should be part of the name, it would be Positive, Negative, Cognitive Symptoms disorder. Stakeholder input and linguistic evaluation are essential for successful implementation and global acceptance.

**Supplementary Information:**

The online version contains supplementary material available at 10.1007/s00406-025-02018-8.

## Introduction

Shortly after Emil Kraepelin introduced his classification of severe mental illnesses into manic-depressive insanity and dementia praecox, Eugen Bleuler proposed the term “schizophrenia(s).” Bleuler’s rationale was: First, the course of the disorder is not invariably chronic, as later confirmed by numerous studies, nor do all patients inevitably become ‘demented’. By using the plural form, he anticipated that schizophrenia would not be a singular, uniform condition but rather a spectrum of disorders. Additionally, the absence of a suitable adjectival form derived from “dementia praecox” influenced his choice. However, Bleuler primarily favoured the term"schizophrenia"because he considered ‘the splitting of various mental functions’ to be its defining characteristic.

Etymologically,"schizophrenia"originates from Greek and literally translates to “splitting of the diaphragm”, reflecting the ancient Greek belief that the diaphragm was the seat of the soul. In the early twentieth century, there was a trend towards adopting Greek terminology in an effort to render psychiatry more ‘scientific’ (e.g., abulia, catatonia, dysphoria). However, the term itself carries an enigmatic quality that may contribute to the stigmatization of those affected. The public often misinterprets schizophrenia as meaning ‘split personality’, and continues to associate the disorder with dangerousness, unpredictability, and other negative traits [1]. Surveys indicate that both individuals with lived experience and healthcare professionals find the term stigmatizing [2]. While renaming schizophrenia is unlikely to eradicate stigma entirely, it may help reduce it.

A second rationale for renaming schizophrenia is that the concept of ‘splitting of mental functions’ does not reflect the current conceptualisation of the disorder. According to the ICD-11, schizophrenia is characterized by *disturbances* across multiple domains—including thought, perception, self-experience, cognition, affect, behavior, and psychomotor function. Similarly, the DSM-5-TR describes it as a *disorder* of “cognitive, behavioral, and emotional dysfunctions.” These descriptions emphasize *disturbances* and *dysfunctions* rather than a *splitting* of mental functions.

Critics of a name change argue that the term “schizophrenia” is well established and that its renaming might trivialize a severe condition. While we agree that a new name should not sound belittling, this argument does not justify retaining a stigmatizing term that fails to adequately describe the disorder. Notably, preliminary experiences with renaming “schizophrenia” in Asia were positive [3]. Psychiatry has also seen other, overall successful, name changes, such as replacing “manic depressive illness” with “bipolar disorder,” “hysteria” with “conversion disorder”, and “mental retardation” with “intellectual disability.”

## Requirements for a new name

### Translatability

The new term should be readily translatable into multiple languages. Inconsistent nomenclature across countries can cause confusion, as seen in certain Asian countries where similar yet distinct terms to replace “schizophrenia” have been developed (Table 1). Some proposed terms, such as the Korean “attunement disorder”, are challenging to translate.

**Table 1:** Evaluation of names which have been proposed for replacing schizophrenia

Proposed Term/Theme	Name Examples/Variations	WHO: NotOffensive or Not Stigmatising	Reflects current ICD/DSM Conceptualisation of Schizophrenia	Name Change for Related Disorders easily feasible	Fits the General Structure of ICD/DSM	Translatable	WHO: Short	WHO Criterion:"Clinical Symptoms"/"Physiology"	WHO: No Name of a Person	WHO: Balance of Science, Communication, Policy
Positive and Negative Symptoms Disorder	PND, Positive-Negative Disorder, Positive-Negative Symptoms Disorder, Positive, Negative Symptoms Disorder, PosiNeg Disorder, PosNeg Disorder	+	+	+	+	+	±	+	+	+
Positive, Negative and Cognitive Symptoms Disorder	Positive and Negative/Cognitive Symptoms Disorder, PNCD	+	±	+	+	+	–	+	+	+
Positive, Negative, Cognitive and Motor Symptoms Disorder	Positive, Negative, and Motor Symptoms Disorder	+	±	+	+	+	–	+	+	+
Disintegration	-Integration Disorder -Disintegration Disorder -Brain Disintegration Disorder -Integrative Disorder -Integrative Mental Disorder -Disintegrative Disorder -Mind Integration Failure Disorder -Neuro-Emotional Integration Disorder (NEID)	±	±	±	±	+	+	±	+	–
Disorganisation/- coordination	-Discoordination Disorder -Disorganized Disorder -Dysfunctional Thought Disorder -Disorganized Thinking Disorder	±	±	±	±	+	+	±	+	–
Connection Problem	Disconnectivity Disorder	+	–	±	–	±	+	±	+	±
Attunement	-Attunement Disorder -Brain Tuning Disorder	+	–	±	–	–	+	–	+	–
Psychosis	-Primary Psychosis -Psychosis Spectrum Disorder -Psychotic Syndrome Psychosis Susceptibility Syndrome (PSS) -Developmental Psychosis -Neurodevelopmental Psychosis -Vulnerability-based Psychosis -Endogenous Psychosis -Nonaffective (enduring) Psychosis -Multi-symptomatic Psychosis and Processing Disorder	–	±	+	–	+	±	+	+	–
Misperception	-Dysfunctional Perception Syndrome -Perception Syndrome -Altered Reality Syndrome	+	±	±	–	–	±	±	+	–
Detachment	-Detachment Syndrome with Dissociative Features -Chronic Detachment Syndrome	+	±	–	±	–	–	±	+	–
Salience	Salience syndrome	+	–	–	–	–	+	±	+	–
Sensitivity	Sensitive Mind Disorder	±	–	–	–	–	+	–	+	–
Dopamine/Neurotra nsmitter	-Dopamine Syndrome -Dopamine Dysregulation Disorder -Monoamine Dysregulation Disorder	+	–	±	–	+	±	+	+	±
Eponymes	-Kraepelin–Bleuler disease’ -Bleuler’s and Kretschmer’s syndrome -Schneider’s Syndrome -Bleuler’s syndrome -Kraepelin disease -John Nash syndrome	±	–	–	–	+	+	–	–	–
Dysfunction of Perception and Thought	-Disorder with Dysfunction of Thought and Perception -Dysfunction of Thought and Perception -Thought and Perceptual Dysregulation	+	±	±	–	–	–	±	+	–
CONative, COgnitive and Reality Distortion (CONCORD) Syndrome	CONative, COgnitive and Reality Distortion (CONCORD) Syndrome	±	+	±	+	–	±	±	+	±
Neurodevelopmenta l Vulnerability	Neurodevelopmental Vulnerability Disorder	+	–	–	–	+	–	±	+	±
Social Brain Disorder	Social Brain Disorder	+	–	±	–	±	+	–	+	–
Dysphrenia	Dysphrenia	–	–	±	–	±	+	–	+	–
Schizophrenia- related	-Schizophrenic Syndrome -Schizophrenia Spectrum Disorder	–	+	±	+	+	±	–	+	–

+ rather fulfills the criterion, –rather does not fulfill the criterion, ± partially fulfills the criterion; references of the articles in which we found these names can be found in the online appendix

### Stigma reduction

According to WHO guidelines, medical terminology should avoid causing “offense to any cultural, social, national, regional, professional, or ethnic group.” Ideally, the term should carry positive connotations. For instance, ‘sanitation engineer’ has a positive connotation, whereas ‘garbage man’ has a negative one.

### ICD compatibility

The new term must conform to the ICD classification system. For instance, if schizophrenia is renamed, schizoaffective disorder must also be renamed.

### Adherence to WHO naming criteria

The new term should comply with the general rules for disease nomenclature set forth by the World Health Organization (see WHO Name Criteria and ContentModelGuide). The name should balance science, communication and policy. Ideally, it should be concise or, if longer, have a short acronym. The use of personal names is not permitted. For example “Bleuler’s disease” is not applicable. The WHO criteria (available at: WHO Name Criteria) would categorize a name under “Generic Descriptive Terms”, which include:

- Physiological Processes.
- Pathological Abnormality (“reference”).
- Anatomical Systems.
- Clinical Symptoms.

The first three categories are unsuitable for “schizophrenia”. In the early twentieth century, when Alzheimer and Nissl observed pathological changes under their microscopes, they classified the atrtributed the condition to neurology, if not to psychiatry [4]. They put psychiatry on a difficult track. More than a century later, we know that people with “schizophrenia” may exhibit structural brain abnormalities such as diffuse brain volume loss, more pronounced in some areas than others. However, these changes are too subtle to enable diagnosis by routine brain imaging. Similarly, our understanding of the underlying pathophysiology remains incomplete. While dopaminergic neurotransmission plays a role, the precise mechanisms, contributions of other neurotransmitter systems, trauma and the immune system are still to be explored. Consequently, names like “dopamine disorder” fall short of characterising schizophrenia.

At present, the only viable WHO category for schizophrenia is “Clinical Symptoms” (WHO Name Criteria). Ideally, if the essential feature or core of schizophrenia could be identified, it could serve as a name. Large-scale qualitative studies of patients’ experiences could provide valuable insights into “core” features of the disorder and should be conducted, but there is a risk of tautology—individuals diagnosed with schizophrenia are likely to report positive symptoms during acute phases and negative and cognitive symptoms during stable phases.

Therefore, it is necessary to derive a new name that aligns with the current conceptualization of schizophrenia in the ICD-11 and DSM-5-TR. Both diagnostic frameworks—referred to as “essential (required) features” in the ICD-11 and “diagnostic criteria” in the DSM-5-TR—include positive symptoms (delusions, hallucinations, disorganised speech/thought), negative symptoms, and psychomotor disturbances. However, we propose excluding psychomotor disturbances from the new name, as these are most characteristic of catatonia, which is recognized as a separate disorder in the ICD-11 and serves as a cross-diagnostic “specifier” in the DSM-5-TR. Although cognitive dysfunction is common and often severe in schizophrenia – on average about 1.5 standard deviations below that of controls [5]—cognition is mentioned only in the accompanying text, not within the diagnostic criteria in both the ICD-11 and DSM-5-TR. The DSM-5 group has justified this decision in that cognitive deficits are not unique to schizophrenia; they also occur in other disorders, such as major depression and bipolar disorder, albeit to a lesser extent [7]. Some patients with schizophrenia preserve cognition [6]. Although cognitive deficits and negative symptoms are considered distinct issues, they are strongly correlated. Negative symptoms, such as lack of motivation and low energy, significantly impact on cognitive performance [7]. Moreover, from a linguistic perspective, the term “deficit” implies a form of “negativity.” Thus, aligning with the WHO requirement for brevity, we suggest to subsume cognitive deficits under negative symptoms. However, these considerations do not mean that cognitive deficits, similar to psychomotor disturbances, cannot become part of the diagnostic criteria in ICD-12 and DSM-6. On the contrary, we believe they should be included.

## Evaluation of current proposals

A PubMed search with the term (schizophren* OR psychos* OR psychotic*) AND (name* OR nami* OR renam* OR nomen* OR notion* OR term* OR replace*) NOT long*term NOT terminal NOT psychosocial NOT termination NOT short-term yielded multiple alternative names which we categorized and evaluated their alignment with the aforementioned requirements (see Table 1 and eTable 1). Some examples:

The East Asian terms (e.g., Japan: Togo-Shitcho-Sho “Integration Disorder”; Taiwan: Sī jué shī tiáo zhèng “dysregulation of thought and perception”; South Korea: Johyeon-byeong “attunement disorder”) are based on Bleuler’s definition, yet avoid the term ‘split’. It remains uncertain whether Bleuler’s concept of a ‘splitting apart’ of mental functions accurately encapsulates the core nature of schizophrenia.

Terms emphasizing ‘dysfunctional perception’ or ‘salience’ are deficient, as they only address positive symptoms while neglecting negative symptoms.

Terms based on “psychosis” are problematic. Psychosis is part of the chapter title in both the DSM-5-TR (Schizophrenia Spectrum and Other Psychotic Disorders) and ICD-11 (Schizophrenia or Other Primary Psychotic Disorders). It is thus a higher level construct. Psychosis occurs also in major depression, delirium, autism, and bipolar disorder. In other words, psychosis is a syndrome which can occur in several instances, “schizophrenia” is one possible manifestion of psychosis. Thus, replacing “schizophrenia” with “psychosis” would not be compatible with the current ICD system (WHO requirement 3). Furthermore, a term based on psychosis may not reduce stigma, because – as schizophrenia—it can be adjectivized (e.g., “that is so psychotic/schizophrenic”); a point on which we disagree with Bleuler, who considered adjectivation beneficial.

The term CONCORD (Conative, Cognitive, and Reality Distortion Syndrome) has been proposed; however, its associations with aviation (as in the Concorde airplane) and uncertainty whether “schizophrenia” is consistently conative render it suboptimal.

## Positive and Negative Symptoms Disorder (PND)

We propose the term **Positive and Negative Symptoms Disorder** (PND) which meets the requirements stated above. This designation explicitly describes the symptomatology of schizophrenia and is easily translatable. Many psychiatric disorders are named based on their primary symptoms (e.g., panic disorder, obsessive–compulsive disorder, attention-deficit/hyperactivity disorder, depressive episode, manic episode). The term is neutral and unlikely to contribute to stigma. Notably, starting with “positive” creates an initially favorable impression for laypeople. When further explained—that positive symptoms refer to hallucinations, delusions and disorganized thought/speech —it also allows for discussion of how, in some cases, these symptoms may be associated with creativity [9]. For reasons of brevity we tend to subsume cognitive dysfunction under negative symptoms. In the form of formal thought disorder they are also part of positive symptoms. However, we support future iterations of ICD and DSM incorporating cognitive deficits as a more defining feature into the diagnostic criteria, given the substantial evidence supporting their role. This aligns with Kraepelin’s original characterization of schizophrenia as “*dementia praecox”*. The proposed name is consistent with the ICD structure (ContentModelGuide; as per WHO requirement 3). Alternatively, the name could be Positive, Negative, Cognitive Symptoms Disorder. If motor symptoms should still be part of it, it would be Positive, Negative, Motor Symptoms Disorder or Positive, Negative, Cognitive, Motor Symptoms Disorder.

## Implications for related diagnoses

Renaming schizophrenia necessitates corresponding changes to related disorders.

- Schizoaffective disorder would be renamed “Positive, Negative, Affective Symptoms Disorder (PNAD)”. Again, if cognitive symptoms were part of the name, it would become unpronouncably long (positive, negative, cognitive, affective symptoms disorder).

- Delusional disorder might remain unchanged, as delusions are its defining feature.

- Catatonia (ICD-11) remains unchanged.

- Schizotypal disorder—“Subthreshold PND”.

- For schizophreniform disorder (DSM-5-TR)/acute and transient psychotic disorder (ICD-11), we propose “Brief PND”. A decision must be made whether DSM-5-TR’s Brief Psychotic Disorder should be merged with Schizophreniform Disorder. This would require shortening the diagnostic duration for schizophrenia from six months to one month, aligning the DSM classification with ICD-11 and simplifying its use in clinical practice.

## “Disorder” *versus* “disease” and “syndrome”

As with almost all psychiatric diagnoses in ICD and DSM, we recommend using “disorder” rather than “disease” (which implies a biological cause), “condition” (which does not translate across languages, e.g. German), or “syndrome”. If syndrome were chosen, most psychiatric disorders would have to be renamed syndromes. But a syndrome is a higher-order construct. It is a group of signs and symptoms which occur together (e.g. depressive syndrome, manic syndrome). Their exact composition, which symptoms are most prominent, duration etc defined the lower-level, more specific construct, the disorder. In this framework, “psychosis” would remain an overarching syndrome, with PND representing one of its manifestations.

Finally, this proposal aligns with recent recommendations suggesting that psychiatric classifications should prioritize symptoms over higher-order constructs [10]. Psychiatric symptoms are indisputable and “nearer to nature”, whereas diagnostic constructs in psychiatry are consensus-based and thus prone to criticism. Moreover, most psychiatric treatments target symptoms regardless of the underlying disorder [10]. The approach follows the opinion voiced by many clinicians in a Swiss survey who advocated for naming “schizophrenia” based on its symptoms (A. Maatz personal communication).

## Next steps

Ultimately, the decision to rename schizophrenia rests with the relevant stakeholders, including patients and their families, experts in the field (e.g., members of the Schizophrenia International Research Society [SIRS], Society for Research in Psychopathology, APA, EPA, ECNP, CINP, national psychiatric associations etc.), representatives from the ICD and DSM, and linguistic specialists. Most importantly, individuals with lived experience should be included in the decision-making process, as they are directly affected by any changes and bring invaluable insights.

## Supplementary Information

Below is the link to the electronic supplementary material.

Supplementary file1 (XLSX 24 KB)

## Data Availability

No data was used for the research described in the article.

## References

[1] Schomerus G et al (2012) Evolution of public attitudes about mental illness: a systematic review and meta-analysis. Acta Psychiatr Scand 125:440–452. 10.1111/j.1600-0447.2012.01826.x 22242976 10.1111/j.1600-0447.2012.01826.x

[2] Mesholam-Gately RI et al (2021) Are we ready for a name change for schizophrenia? A survey of multiple stakeholders. Schizophr Res 238:152–160. 10.1016/j.schres.2021.08.034 34688117 10.1016/j.schres.2021.08.034

[3] Yamaguchi S et al (2017) Associations between renaming schizophrenia and stigma-related outcomes: a systematic review. Psychiatry Clin Neurosci 71:347–362. 10.1111/pcn.12510 28177184 10.1111/pcn.12510

[4] Shorter E (1998) A history of psychiatry: from the era of the asylum to the age of prozac. Wiley, Cham.

[5] McCutcheon RA, Keefe RSE, McGuire PK (2023) Cognitive impairment in schizophrenia: aetiology, pathophysiology, and treatment. Mol Psychiatry 28:1902–1918. 10.1038/s41380-023-01949-9 36690793 10.1038/s41380-023-01949-9 PMC10575791

[6] Oomen PP et al (2022) The neurobiological characterization of distinct cognitive subtypes in early-phase schizophrenia-spectrum disorders. Schizophr Res 241:228–237. 10.1016/j.schres.2022.02.006 35176721 10.1016/j.schres.2022.02.006

[7] Zanelli J et al (2010) Specific and generalized neuropsychological deficits: a comparison of patients with various first-episode psychosis presentations. Am J Psychiatry 167:78–85. 10.1176/appi.ajp.2009.09010118 19952077 10.1176/appi.ajp.2009.09010118

[8] Harvey PD (2011) Mood symptoms, cognition, and everyday functioning: in major depression, bipolar disorder, and schizophrenia. Innov Clin Neurosci 8:14–18 22132366 PMC3225134

[9] Kaufman SB, Paul ES (2014) Creativity and schizophrenia spectrum disorders across the arts and sciences. Front Psychol 5:1145. 10.3389/fpsyg.2014.01145 25404921 10.3389/fpsyg.2014.01145 PMC4217346

[10] Leucht S, van Os J, Jager M, Davis JM (2024) Prioritization of psychopathological symptoms and clinical characterization in psychiatric diagnoses: a narrative review. JAMA Psychiat 81:1149–1158. 10.1001/jamapsychiatry.2024.2652 10.1001/jamapsychiatry.2024.2652 39259534

